# Antimetastatic Effects of *Ganoderma lucidum* Polysaccharide Peptide on B16-F10-luc-G5 Melanoma Mice With Sleep Fragmentation

**DOI:** 10.3389/fphar.2021.650216

**Published:** 2021-07-08

**Authors:** Haocheng Xian, Jiayi Li, Yimeng Zhang, Ditian Li, Yinan Zhu, Siyan Li, Zhelun Tan, Zhibin Lin, Xuejun Li, Yan Pan

**Affiliations:** Department of Pharmacology, School of Basic Medical Sciences, Health Science Center, Peking University and Beijing Key Laboratory of Tumor Systems Biology, Peking University, Beijing, China

**Keywords:** *Ganoderma lucidum* polysaccharide peptide, sleep fragmentation, tumor metastasis, proteomics, gut microbiota

## Abstract

*Ganoderma lucidum* (Lingzhi) polysaccharide peptide (GL-pp) is a component of the globally acknowledged traditional Chinese medicine *Ganoderma lucidum*; *Ganoderma lucidum* is known for its sedative, hypnotic, immune regulatory, antitumor, and other pharmacological effects. In recent years, sleep disorders have been linked to many diseases and human body disorders, including cancer. Some experimental studies in mice found that sleep fragmentation could promote tumor development and progression. However, effects on GL-pp on tumor metastasis under circumstances of sleep disorders have rarely been studied. Thus, in this study, we used mice with sleep fragmentation (SF) bearing B16-F10-luc-G5 melanoma tumors to investigate the effect of SF on melanoma metastasis. Furthermore, we investigated the antitumor and antimetastatic effects of GL-pp (80 mg/kg) in mice suffering from SF and bearing B16-F10-luc-G5. Then, whole proteomics was used to analyze the differences in protein expression in the lung tissue between SF mice bearing B16-F10-luc-G5 with and without GL-pp administration. High-throughput pyrosequencing of 16S rRNA was also used to analyze the impact of GL-pp on the gut microbiota composition in SF mice bearing B16-F10-luc-G5. Last, the effects of GL-pp on macrophage polarization and TNF-α serum levels were detected. Collectively, we found that SF significantly facilitated the B16-F10-luc-G5 melanoma tumor metastasis in mice, while GL-pp significantly reduced B16-F10-luc-G5 melanoma tumor metastasis under the condition of SF, in which proteomics and gut microbiota had been changed greatly.

## Introduction

Modern people face great pressure and suffer from anxiety, depression, and other adverse psychological conditions, which lead to sleep disorder of varying degrees. An analysis in China claimed a 15.0% proportion of the pooled insomnia prevalence in 2017 (Cao et al., 2017). Sleep disorders can have extensive adverse effects both physically and mentally; for example, they can promote obesity and impair cognition (Espie, 2002; Wang et al., 2014; André et al., 2019). They are also listed as IARC Group 2A carcinogens, and a number of epidemiological and clinical studies have found that multiple sleep disorders significantly increase the risk of cancer (Davis et al., 2001; Schernhammer et al., 2003; Blask, 2009). Sleep fragmentation (SF) is a common type of sleep disorder generally caused by obstructive sleep apnea (Young et al., 2002); to some extent, it significantly increases the cancer incidence and mortality in younger patients (Campos-Rodriguez et al., 2013; Martínez-García et al., 2014), and this effect has been confirmed in animal experiments (Cao et al., 2015). Moreover, recent clinical studies have indicated that sleep fragmentation strongly correlates with tumor metastasis; for example, a case–control study of 11,412 women indicated that sleep disorders were associated with a significant increase in the presence of breast cancer metastases (Jacob et al., 2018). Sleep disorder is also a prominent problem of cancer patients (Lee et al., 2004), and cancer might cause or aggravate sleep disorder. An epidemiological survey showed that 41% of cancer patients suffered from sleep disorders, with 76% accounting for sleep fragmentation (Davidson et al., 2002). Sleep disorders in cancer patients are mostly caused by worries and cancer pain, and also by medications (Borniger et al., 2015). A previous study indicated that during the chemical therapy, the proportion of sleep disorder reached 43%, which is three times higher than that in the general population (Palesh et al., 2010). The correlation of sleep fragmentation and cancer further implied the importance of considering sleep fragmentation in tumor-related research.

*Ganoderma lucidum* (Lingzhi) is a multifunctional Chinese herbal medicine and has been extensively studied for the last 20 years. It has a wide range of pharmacological activities, including immunomodulatory effect (Xu et al., 2011), antitumor effect (Zhao et al., 2010), neuropharmacological effect (Cui et al., 2019), and protective effect on the cardiovascular system (Xie et al., 2016). Several studies have shown the inhibitory effects of *Ganoderma lucidum* on tumor growth and metastasis *in vivo* (Loganathan et al., 2014; Lin and Hsu, 2016) and its inhibitory effects on cell migration *in vitro* (Liang et al., 2015; Zhao et al., 2018a). *Ganoderma lucidum* polysaccharide peptide (GL-pp) is one of the principal active components of *Ganoderma lucidum* and has demonstrated its antitumor function in several kinds of tumors (Cao and Lin, 2004). However, its role in tumor metastasis under SF has not yet been studied. *Ganoderma lucidum* has sedation and hypnosis efficacy, and some modern studies have confirmed that the *Ganoderma lucidum* extract has hypnotic effects in freely moving rats and ICR male mice (Chu et al., 2007; Cui et al., 2012; Feng and Wang, 2019). Hence, in the present study, we used the lung metastasis model of melanoma carcinoma and studied the effect and potential mechanisms of GL-pp on the metastasis of tumors under the SF conditions.

## Materials and Methods

### GL-pp Extracted From *Ganoderma lucidum*

GL-pp was isolated by extracting the fruit body of *Ganoderma lucidum* with boiling water. The content of GL-pp in *Ganoderma lucidum* is 2.33%. It is a polysaccharide peptide with a molecular weight of 512,500, and the ratio of polysaccharides to peptide is 94.84%: 5.16%. The polysaccharides consist of d-rhamnose, d-xylose, d-fructose, d-galactose, and d-glucose linked together by β-glycosidic bonds with molar ratios of 0.549: 3.614: 3.167: 0.556: 6.89. GL-pp is a hazel-colored water-soluble powder, provided by Prof. Shuqian Lin, Fuzhou Institute of Green Valley Bio-Pharm Technology, China.

### Cell Culture

B16-F10-luc-G5 cells were engineered to stably express firefly luciferase (Xenogen Corporation, Alameda, CA). The cells were cultured in DMEM containing 10% heat-inactivated fetal blood serum (FBS), 100 μg/ml streptomycin, and 100 U/mL penicillin in a humidified incubator at 37°C with 5% CO_2_ in air.

### Animal Model and Drug Administration

Six-week-old female BALB/c nude mice, weighing 18–20 g, purchased from the Experimental Animal Center of Peking University (Grade II, Certificate No. 11–00–0,004) were housed under the same conditions (a 12-h light/dark cycle and constant temperature of 24 ± 1°C, free access to food and water) for 7 days of adaptation. The mice were randomly divided into four groups: control group under general conditions with no further treatment (CON group), tumor group with the burden of B16-F10-luc-G5 cells (Tumor group), T + SF group with SF and the burden of B16-F10-luc-G5 cells (T + SF group), and GL-pp group with SF, tumor cells burden, and the administration of 80 mg/kg GL-pp (GL-pp group).

B16-F10-luc-G5 cells (5 × 10^6^ cells/mL per mouse) were injected into the mice through the tail vein. SF was induced by a sweeping pole with an interval of 2 min in the cages during the mouse sleep period (12:00 pm to 16:00 pm) every day. GL-pp (80 mg/kg) was administered orally *via* a gastric probe for 15 days after the inoculation of B16-F10-luc-G5 cells in the GL-pp group.

**FIGURE 1:**
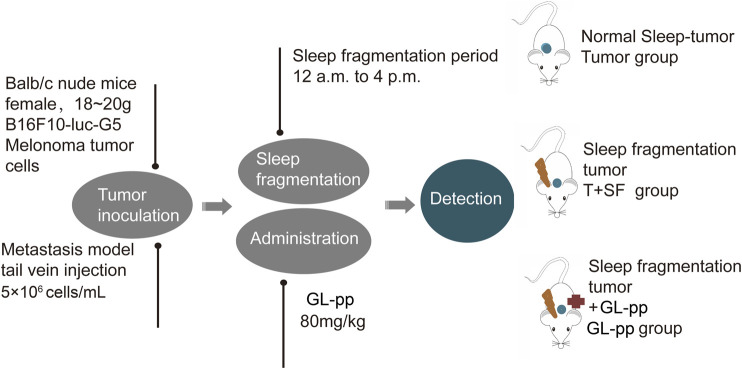
The flowchart of animal treatments.

On day 15, after anesthesia and euthanasia, the lungs, blood serum, and intestines of all mice were harvested for further processing, and the flowchart is shown in Figure 1.

Animal handling and related procedures were approved by the Institutional Animal Care and Use Committee of Peking University Health Science Center.

### *In Vivo* Bioluminescence Imaging

The mice were anesthetized with 1–3% isoflurane (Abbott Laboratories, Chicago, IL), and they received D-luciferin dissolved in phosphate-buffered saline (PBS) at a dosage of 150 mg/kg by intraperitoneal injection on day 8, day 11, and day 15 for bioluminescence imaging. Ten minutes after the injection, the bioluminescence images were collected and measured with the Xenogen IVIS imaging system (Xenogen Corp, Alameda, CA, United States). A region of interest was drawn on each mouse, and the luminescent images were acquired. The resulting gray scale photographic and pseudocolor luminescent images were automatically superimposed by software so that identification of any optical signal with location on the mouse was facilitated. Optical images were displayed and analyzed with the Igor (WaveMetrics, Lake Oswego, OR) and IVIS Living Image (Xenogen) software packages. Regions were manually drawn around the bodies of the mice to assess signal intensity emitted. Optical signal was expressed as photon flux, in units of photons/s/cm2/steradian.

### Lung Histological Observation

The lung tissues were fixed with 4% paraformaldehyde and embedded using paraffin. Paraffin-embedded sections (4 µm) were deparaffinized and stained with hematoxylin and eosin (H&E) using a standard protocol. Five random fields from each sample were taken on each hematoxylin and eosin–stained lung tissue section. The alveolar integrity was evaluated by the area percentage of alveoli in the lung observed by light microscopy with ImageJ software. The mean value was accepted as representative of the sample.

### Sample Preparation for Shotgun Analysis

Lung tissues were flash-frozen in liquid nitrogen and stored at −80°C. Proteins were extracted using RIPA lysis buffer, and each group was analyzed in triplicate.

Protein samples (200 μg) from each group were processed in line with the manufacturer’s protocol (Wiśniewski et al., 2009). Proteins were concentrated using Vivacon 500 filtration tubes (Cat No. VNO1HO2, Sartorius Stedim Biotech, United Kingdom), mixed with 100 μL of 8 M urea in 0.1 M Tris/HCL (pH 8.5), and centrifuged at 14,000 *g*, 20°C, for 15 min. This step was performed twice, after which 10 μL of 0.05 M tris-(2-carboxyethyl) phosphine (TCEP) in water was added, and the samples were incubated at 37°C for 1 h; then, 10 μL of 0.1 M iodoacetamide (IAA) was added, and the samples were incubated in darkness for 30 min. Finally, 4 μg of trypsin (Promega, Madison, WI) in 100 μL of 50 mM NH_4_HCO_3_ was added to each filter. The protein to enzyme ratio was 50:1. The samples were incubated overnight at 37°C, and the released peptides were collected by centrifugation.

**FIGURE 2:**
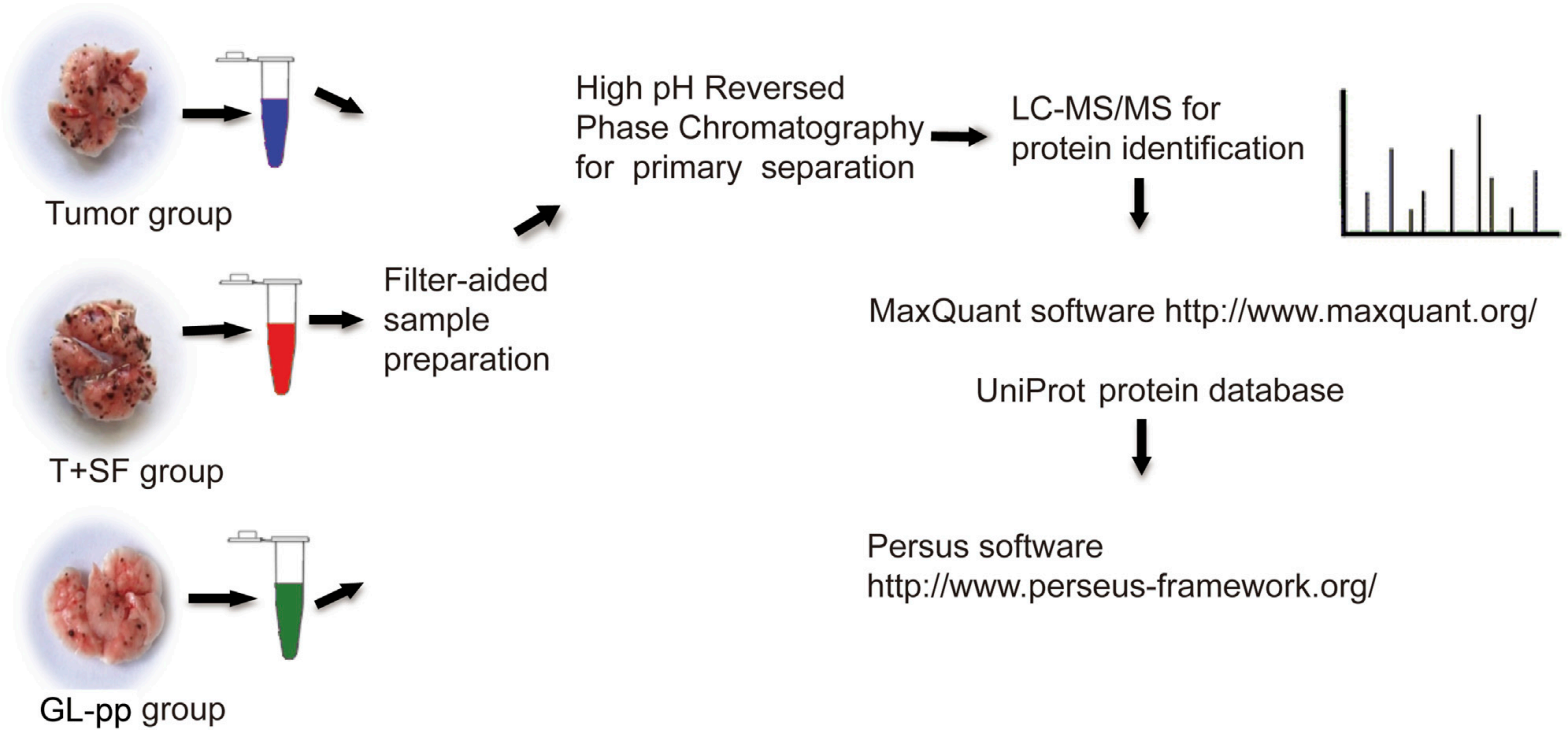
The flowchart of quantitative proteomic analysis.

High-pH reverse-phase chromatography was performed using a Dionex Ultimate 3,000 Micro Binary HPLC Pump system (Yang et al., 2012). The digested peptide mixture was reconstituted with 600 μL buffer A (20 mM ammonium formate in water, pH 10) and loaded onto a 2.1-mm × 150-mm Waters BEH130 C-18 column containing 3.5 μm particles. Peptides were eluted at a flow rate of 230 μL/min with a gradient of buffer B (20 mM ammonium formate in 80% acetonitrile, pH 10): 5% for 5 min, 5%–15% for 15 min, 15%–25% for 10 min, 25%–55% for 10 min, and 55–95% for 5 min. The system was then maintained in 95% buffer B for 5 min before equilibrating with 5% buffer B for 10 min prior to the next injection. Elution was monitored by measuring the absorbance at 214 nm, and fractions were collected every 2 min. The fractions containing eluted peptides were pooled into 15 fractions based on peptide density (Yang et al., 2012) and vacuum-dried for nano-ESI-LC-MS/MS analysis. The related flowchart is shown in Figure 2.

### LC-MS/MS Analysis

LC-MS experiments were performed on a nano-flow HPLC system (Easy-nLC II, Thermo Fisher Scientific, Waltham, MA, United States) connected to an LTQ-Orbitrap Velos Pro (Linear quadrupole ion trap-Orbitrap mass analyzer) mass spectrometer (Thermo Fisher Scientific) equipped with Nanospray Flex Ion Source (Thermo Fisher Scientific). The peptide mixtures (5 μL) were injected onto a pre-column (Easy-column C18-A1, 100 μm I.D. × 20 mm, 5 μm, Thermo Fisher Scientific) at a flow rate of 5 μL/min. Chromatographic separation was performed on a reverse-phase C18 column (Easy-column C18-A2, 75 μm I.D. × 100 mm, 3 μm, Thermo Fisher Scientific) with a 60-min gradient of 2–40% acetonitrile in 0.1% formic acid at a flow rate of 300 nL/min. The electrospray voltage was maintained at 2.2 kV with the capillary temperature at 250°C. The LTQ-Orbitrap was operated in a data-dependent mode to simultaneously measure full-scan MS spectra (*m/z* 350–2000) in the Orbitrap with a mass resolution of 60,000 at *m/z* 400. After the full-scan survey, 15 most abundant ions detected in the full-MS scan were measured in the LTQ-Orbitrap using collision-induced dissociation (CID).

### Protein Identification and Quantitation

Data analysis was performed with MaxQuant software (v1.4.1.2). For protein identification, MS/MS data were submitted to the UniProt human protein database (release 3.43, 72,340 sequences) using the Andromeda search engine (Cox et al., 2011) with the following settings: trypsin cleavage, fixed modification of carbamidomethylation of cysteine, variable modifications of methionine oxidation, a maximum of two missed cleavages, and a false discovery rate calculated by searching the decoy database. Other parameters were set as default. The results were imported into Microsoft Excel for further analysis.

Label-free quantitation (LFQ) was also performed in MaxQuant. The minimum ratio count for LFQ was set to 2, with the match-between-runs option enabled. Other parameters were set as default. Upregulated and downregulated proteins were defined based on a significantly altered protein ratio (*p* < 0.05).

### Global Signal Transduction Network

The global signal transduction networks that had been experimentally validated were used for the observation of protein–protein and protein–DNA interactions. From the KEGG database, we were able to illustrate the interaction between differentially expressed genes (Jia et al., 2011). The nodes in the network were connected when their corresponding encoded gene products were connected directly or indirectly by a linker gene in the interaction network. The importance of each gene was measured by counting the number of upstream, downstream, or binding genes, which were shown as in-degree, out-degree, or degree, respectively. The degree was defined as the link number of one node with others, and genes with higher degrees occupied more important positions in the signaling network. *p* < 0.05 was considered statistically significant.

### Functional Enrichment Analysis

Gene Ontology (GO) Biological Process and Kyoto Encyclopedia of Genes and Genomes (KEGG) pathway enrichment analysis were performed on Metascape (https://metascape.org/) (Zhou et al., 2019). All genes in the genome were used as the enrichment background. Terms with a *p*-value less than 0.01, an enrichment factor over 1.5, and a minimum count of three were grouped into clusters based on the membership similarities. Kappa scores were used as the similarity metric, and the similarity threshold of sub-trees was 0.3. The most statistically significant term within a cluster was chosen to represent the cluster. *p*-values were calculated based on the accumulative hypergeometric distribution, and the Benjamini–Hochberg procedure was used for multiple test correction.

A subset of enriched terms was selected and rendered as a network plot, and the terms with a similarity over 0.3 were connected by edges. The terms were selected with the best *p*-values from each of the 20 clusters and with the constraint of no more than 15 terms per cluster and no more than 250 terms in total. The network was visualized using Cytoscape (Shannon et al., 2003), where each node represented an enriched term and was colored by its cluster ID.

### Microbial Sequencing

The DNA from the intestinal content was isolated with a QIAamp DNA Stool Mini Kit (Qiagen) following the manufacturer’s instructions. The diluted samples were then PCR-amplified using bar-coded primers, including 16S V4 (515F and 806R), V3–V4, V4–V5, and V5–V7 regions. The Ion Plus Fragment Library Kit (48 rxns, Thermo Fisher Scientific) was used to build the library, and after Qubit quantification and library test, the Ion S5 XL System (Thermo Fisher Scientific) was used for sequencing. The low-quality parts of reads were cut off using Cutadapt (v1.9.1) (Martin, 2011), and then the sample data were split from the reads according to the barcode to get raw reads. The chimeric sequence was identified compared with the species annotation database (https://github.com/torognes/vsearch/) and removed to obtain the final effective data (clean reads) (Haas et al., 2011). The clean reads were clustered to operational taxonomic units (OTUs) at a 97% identity using UPARSE software (v7.0.1001) (Edgar, 2013). The taxonomic information was obtained with the Mothur method and the SSUrRNA database of SILVA132 (Quast et al., 2012) for species annotation analysis, and all data were homogenized based on the sample with the least data. Alpha and beta diversity indexes were calculated using QIIME software (v1.9.1), and the LDA threshold was set at four to perform LDA effect size (LEfSe) analysis. The taxa with significant differences were identified with the *T* test and the Wilcoxon test.

### Flow Cytometry

The tissue (1 mm^3^ blocks) was added to a tissue grinder with 1 ml saline and homogenate. Then, 10 ml saline was added into the lung homogenate, and cell suspension was collected and filtered with a 300-mesh nylon filter. The fluorescence-labeled PE anti-CD86 (BioLegend, San Diego, CA) or PE anti-CD206 (BioLegend) antibodies and FITC anti-F4/80 antibody (Abcam) were added and incubated at 37°C for 1 h. Samples were then washed twice using PBS and analyzed on an FACS Calibur (BD Biosciences).

### Elisa Experiment

Blood samples were obtained from each mouse and allowed to clot at room temperature for 30 min and centrifuged for 10 min at 3,000 rpm to get blood serum. The serum TNF-α level was tested using a tumor necrosis factor-alpha assay kit (DE5101-96T, BioDee Biotechnology, Beijing, China) in accordance with the manufacturer’s instructions. Optical density (OD) of each well was detected at the wavelength of 450 nm within 15 min, and three replicates were repeated.

### Statistical Analysis

Data were expressed as mean ± standard deviation (SD). Differences between groups were analyzed with multi-factor analysis of variance (multi-way ANOVA, MANOVA), followed by the Tukey comparison test with GraphPad Prism software (San Diego, CA), unless otherwise stated. Statistical significance was defined as *p* < 0.05.

## Results

### GL-pp Abated the Acceleration Effect of SF on Tumor Metastasis in Mice Bearing B16-F10-luc-G5.

**FIGURE 3:**
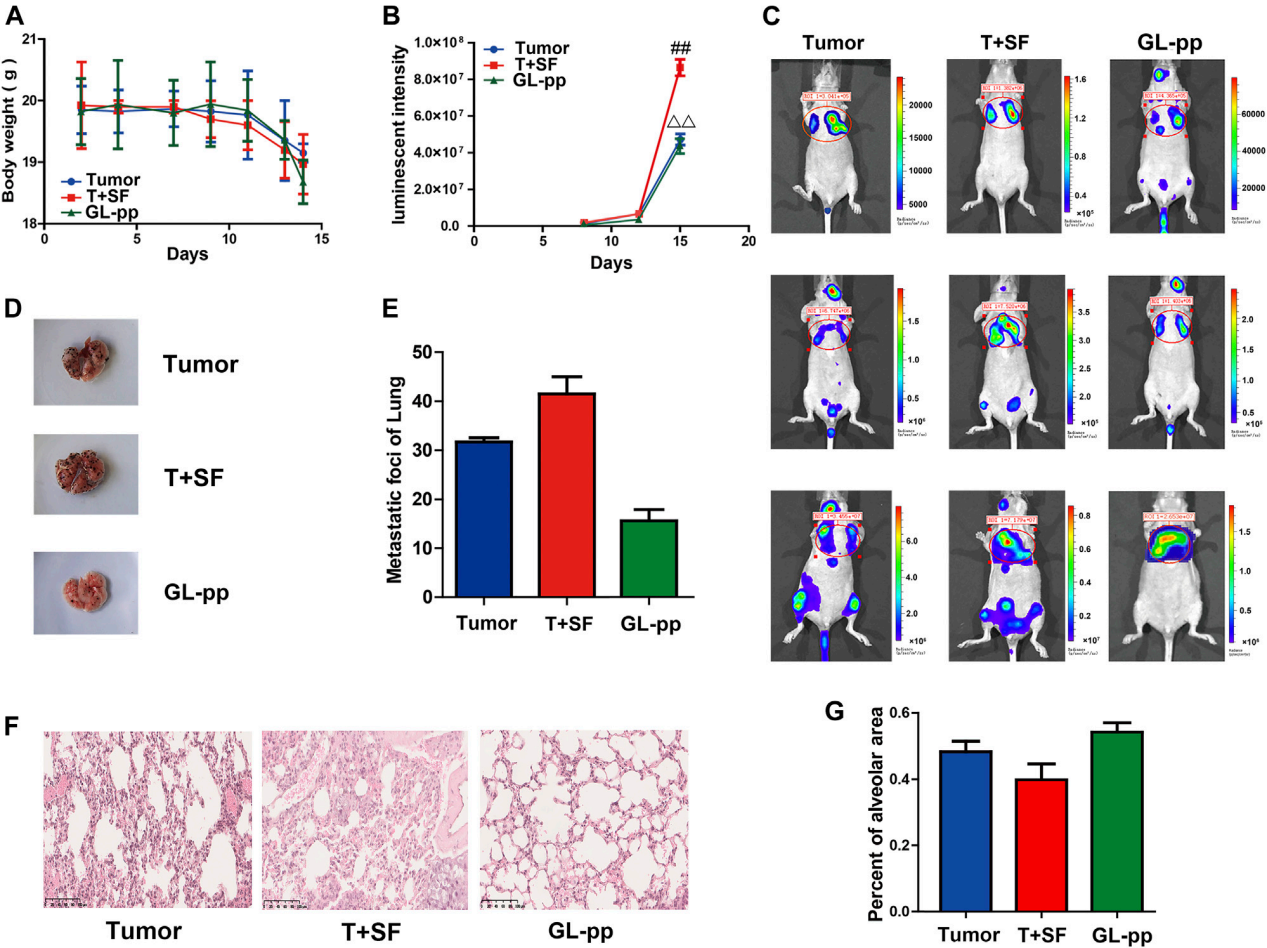
Inhibitory effects of GL-pp on tumor metastasis in mice with B16-F10-luc-G5 and SF. To show relative changes of tumor lung metastasis over time, luminescent intensity images were processed using living image *in vivo*. There were three groups, including tumor group with the burden of B16-F10-luc-G5 cells (Tumor group), T + SF group with SF and the burden of B16-F10-luc-G5 cells (T + SF group), and GL-pp group with SF, tumor cells burden, and the administration of 80 mg/kg GL-pp (GL-pp group). **(A)** The body weight of mice was observed over time. **(B)** The quantification of pulmonary luminescent intensity was recorded with living image *in vivo*. **(C)** Lung metastasis luminescent intensity images of a representative mouse from each group are shown. **(D)** Delegated mice pulmonary tissue with B16-F10-luc-G5 tumor foci of each group is shown. **(E)** The number of lung metastatic foci was counted. **(F)** H&E staining of pulmonary tissue sections (×100). **(G)** Percentage of alveolar area was calculated. The results are shown as mean ± SD, n = 5. ^##^ *p* < 0.01 compared with the Tumor group; ^△△^ *p* < 0.01 compared with the T + SF group.

The tumor metastasis was reflected by *in vivo* imaging as the lung luminescent intensity and the number of lung metastatic foci. The survival rate and body weight were used to reflect the overall survival status (Figure 3). The survival rate was equal in all groups (100%), while the body weight in the T + SF group was lighter than that in the Tumor group. *In vivo* imaging showed significantly stronger lung luminescence intensity in the T + SF group than in the Tumor group (*p* < 0.05) on day 15. Moreover, the number of lung metastatic foci in the T + SF group was greater than that in the Tumor group. As for the GL-pp group, body weight was similar to that in the Tumor group and higher than that in the T + SF group. The *in vivo* lung luminescence intensity decreased significantly compared with the T + SF group (*p* < 0.05), while the number of lung metastatic foci decreased compared with that in the T + SF and Tumor groups.

Consistent with the differences in lung luminescence intensity and metastatic foci, the pulmonary alveolar integrity in the T + SF group was a little worse than that in the Tumor group. Compared with the T + SF group and the Tumor group, the pulmonary alveolar integrity in the GL-pp group was better, indicating a protective effect of GL-pp on lung alveolar integrity in mice burdened with B16-F10-luc-G5 cells suffering from SF (Figures 3F, G).

### GL-pp Altered the Protein Expression of Lung Metastatic Tissue in Tumor-Bearing Mice Subjected to SF and the Related GO and KEGG Pathway Analyses.

To identify the influence of GL-pp on lung tissue metastasis protein expression profiling, we detected the differentially expressed proteins between the GL-pp group and the T + SF group by a label-free quantitative whole proteomics approach. The datasets presented in this study can be found in online repositories (ProteomeXchange Consortium *via* the PRIDE partner repository with the dataset identifier PXD025171).

**FIGURE 4:**
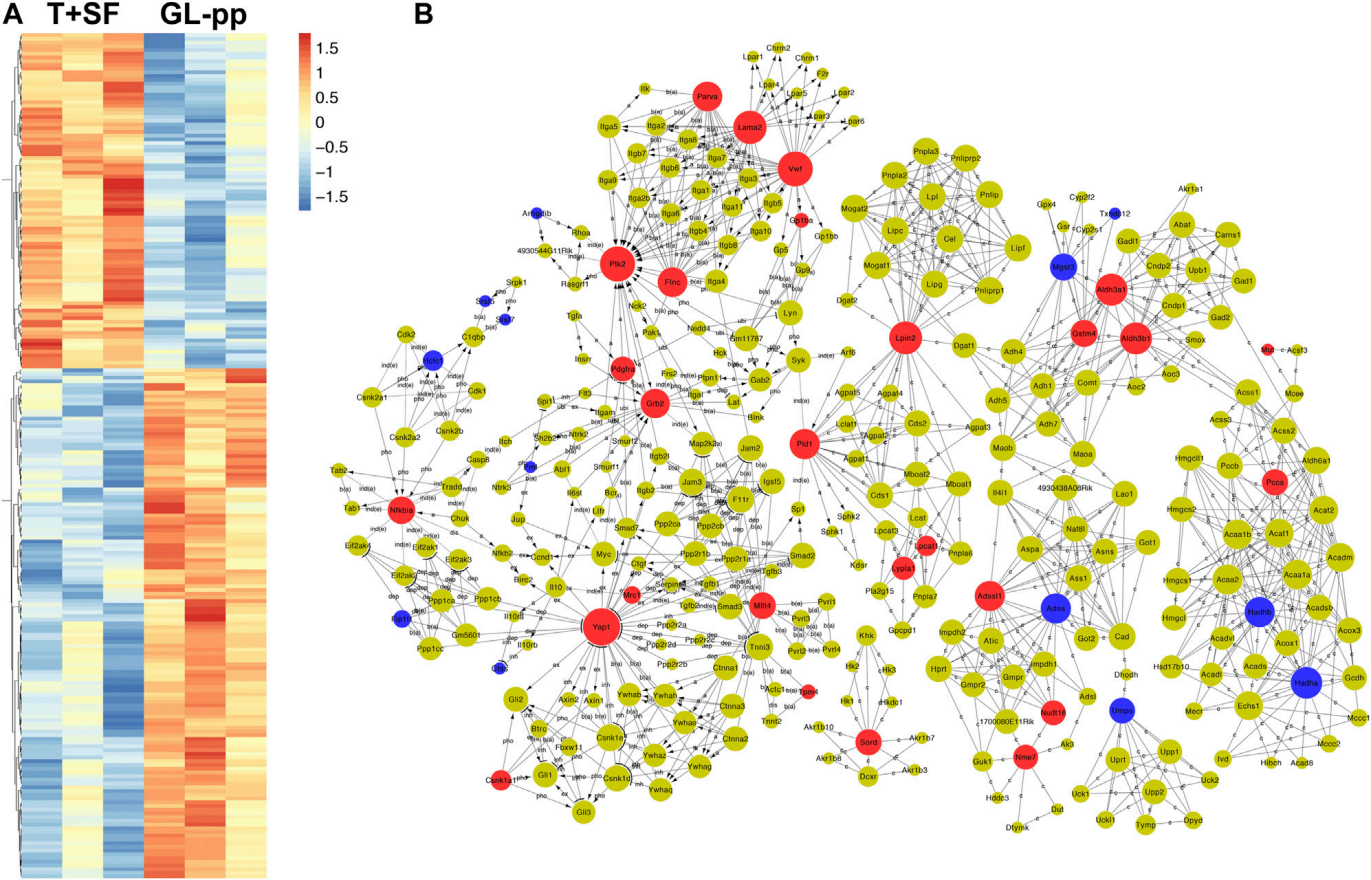
Quantitative protein expression profiling and the global signal transduction network of the significantly altered genes between the T + SF group and the GL-pp group. **(A)** Heat map of 227 significantly differentially expressed proteins between the T + SF group and the GL-pp group with the gene names listed on the right (orange indicates a higher expression level, and blue indicates a lower expression level, *p* < 0.05). **(B)** The global signal transduction network, the nodes in which were connected when their corresponding encoded gene products were connected directly or indirectly by a linker gene in the interaction network, and the size of each node indicates the degree of the gene. Red indicates upregulated and blue means downregulated key regulatory genes.

In total, 227 significantly differentially expressed (upregulated and downregulated) genes were identified at the *p* value of 0.05 (Supplementary Table S1). The heat map of the 227 genes is shown in Figure 4A, including 137 upregulated genes and 90 downregulated genes.

**FIGURE 5:**
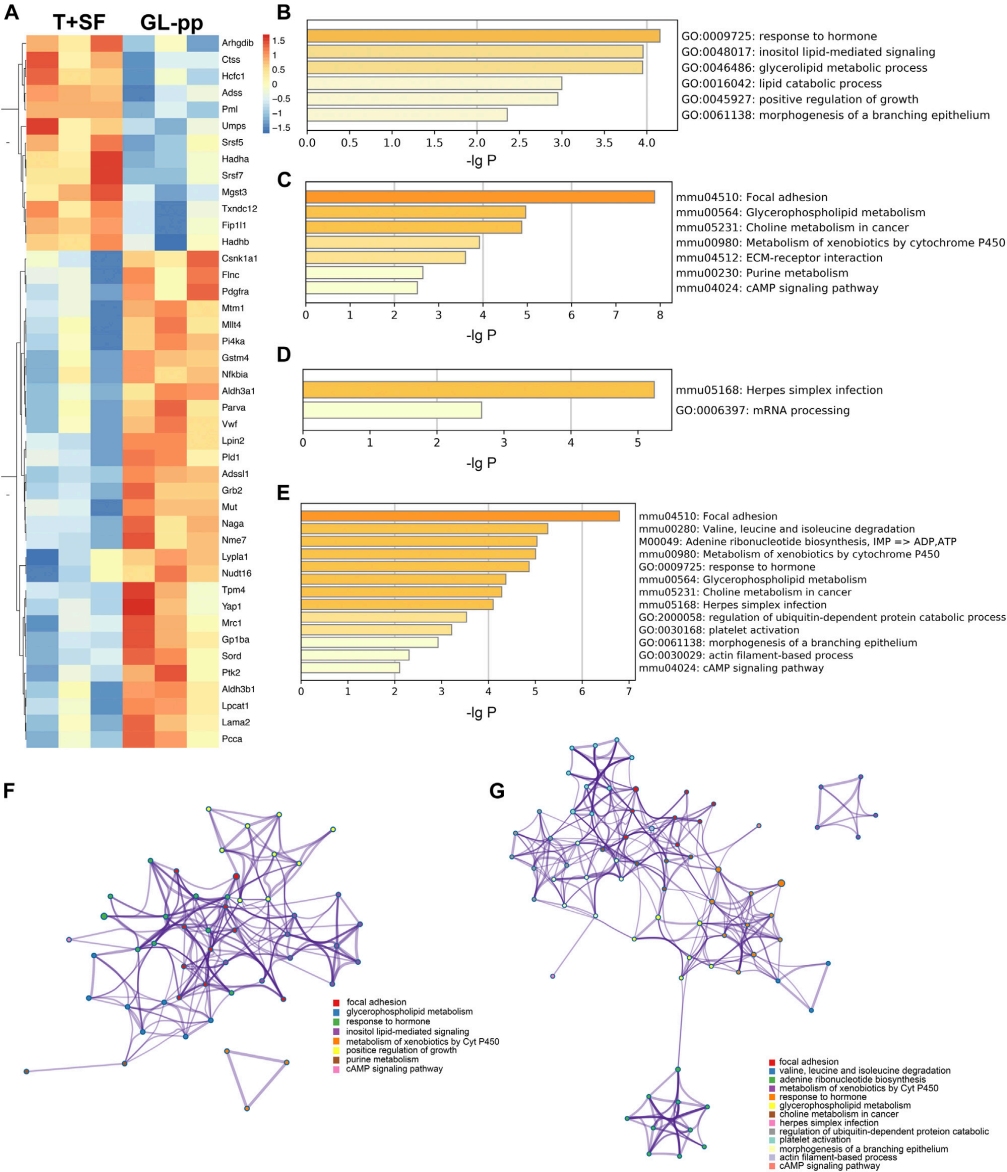
The key regulatory genes between the T + SF group and the GL-pp group and the function enrichment analysis. **(A)** Heat map of 43 key regulatory genes between the T + SF group and the GL-pp group with the gene names listed on the right (orange indicates a higher expression level, and blue indicates a lower expression level). **(B)** The significantly upregulated GO biological process clusters with upregulated different genes. **(C)** The significantly upregulated KEGG pathway clusters with upregulated different genes. **(D)** Significantly downregulated GO biological process and KEGG pathway clusters with downregulated difference genes. **(E)** Significantly altered GO biological process and KEGG pathway clusters among all 43 key regulatory genes. **(F)** The network plot of significantly upregulated pathways was visualized with Cytoscape software. **(G)** The combination of significantly altered pathways was visualized with Cytoscape software. Each node represented an enriched term, and nodes were colored by clusters, that is, the same color in the network means the same; for example, red represents focal adhesion.

The global signal transduction networks that had been experimentally validated were used for the observation of protein–protein and protein–DNA interactions. We performed gene network analysis based on the KEGG signaling pathway influenced by the 227 differentially expressed genes (Figure 4B). The importance of each gene was measured by counting the number of upstream, downstream, or binding genes, which were shown as in-degree, out-degree, or degree, respectively. From the network, 43 key regulatory genes were identified (Table 1), including 30 upregulated genes (*Lama*2, Vwf, Ptk2, Yap1, Aldh3a1, Aldh3b1, Lpin2, Grb2, Pld1, Adssl1, Flnc, Parva, Nfkbia, Sord, Gstm4, Mllt4, Pcca, Mtm1, Nme7, Nudt16, Pdgfra, Lypla1, Tpm4, Csnk1a1, Lpcat1, Gp1ba, Mrc1, Naga, Mut, and Pi4ka) and 13 downregulated genes (Hadha, Hadhb, Adss, Mgst3, Umps, Hcfc1, Fip1l1, Pml, Arhgdib, Ctss, Srsf5, Srsf7, and Txndc12). The heat map of the 43 key regulatory genes is shown in Figure 5A. Genes with higher degrees occupied more important positions in the signaling network.

**TABLE 1:** Forty-three key regulatory genes identified from the global signal transduction network between the T + SF group and the GL-pp group.

protein	Gene symbol	Description	Style	Degree
LAMA2	*Lama*2	Laminin, alpha 2	Up	55
VWF	Vwf	von Willebrand factor homolog	Up	51
FAK1	Ptk2	PTK2 protein tyrosine kinase 2	Up	43
YAP1	Yap1	Yes-associated protein 1	Up	35
AL3A1	Aldh3a1	Aldehyde dehydrogenase family 3, subfamily A1	Up	29
AL3B1	Aldh3b1	Aldehyde dehydrogenase 3 family, member B1	Up	29
LPIN2	Lpin2	Lipin 2	Up	25
GRB2	Grb2	Growth factor receptor bound protein 2	Up	22
PLD1	Pld1	Phospholipase D1	Up	21
PURA1	Adssl1	Adenylosuccinate synthetase like 1	Up	20
FLNC	Flnc	Filamin C, gamma	Up	18
PARVA	Parva	Parvin, alpha	Up	18
IKBA	Nfkbia	Nuclear factor of kappa light polypeptide gene enhancer in B-cell inhibitor, alpha	Up	14
DHSO	Sord	Sorbitol dehydrogenase	Up	14
GSTM4	Gstm4	Glutathione S-transferase, mu 4	Up	12
AFAD	Mllt4	Myeloid/lymphoid or mixed-lineage leukemia (trithorax homolog, drosophila)	Up	11
PCCA	Pcca	Propionyl-coenzyme a carboxylase, alpha polypeptide	Up	11
MTM1	Mtm1	X-linked myotubular myopathy gene 1	Up	9
NDK7	Nme7	NME/NM23 family member 7	Up	7
NUD16	Nudt16	Nudix (nucleoside diphosphate–linked moiety X)-type motif 16	Up	7
PGFRA	Pdgfra	Platelet-derived growth factor receptor, alpha polypeptide	Up	7
LYPA1	Lypla1	Lysophospholipase 1	Up	6
TPM4	Tpm4	Tropomyosin 4	Up	6
KC1A	Csnk1a1	Casein kinase 1, alpha 1	Up	5
PCAT1	Lpcat1	Lysophosphatidylcholine acyltransferase 1	Up	5
GP1BA	Gp1ba	Glycoprotein 1b, alpha polypeptide	Up	4
MRC1	Mrc1	Mannose receptor, C type 1	Up	4
NAGAB	Naga	N-acetyl galactosaminidase, alpha	Up	4
MUTA	Mut	Methylmalonyl-coenzyme a mutase	Up	3
PI4KA	Pi4ka	Phosphatidylinositol 4-kinase, catalytic, alpha polypeptide	Up	2
ECHA	Hadha	Hydroxyacyl-coenzyme a dehydrogenase/3-ketoacyl-coenzyme a thiolase/enoyl-coenzyme a hydratase (trifunctional protein), alpha subunit	Down	27
ECHB	Hadhb	Hydroxyacyl-coenzyme a dehydrogenase/3-ketoacyl-coenzyme a thiolase/enoyl-coenzyme a hydratase (trifunctional protein), beta subunit	Down	21
PURA2	Adss	Adenylosuccinate synthetase, non-muscle	Down	20
MGST3	Mgst3	Microsomal glutathione S-transferase 3	Down	12
UMPS	Umps	Uridine monophosphate synthetase	Down	9
HCFC1	Hcfc1	Host cell factor C1	Down	5
FIP1	Fip1l1	FIP1-like 1 (*S. cerevisiae*)	Down	4
PML	Pml	Promyelocytic leukemia	Down	4
GDIR2	Arhgdib	Rho, GDP dissociation inhibitor (GDI) beta	Down	2
CATs	Ctss	Cathepsin S	Down	2
SRSF5	Srsf5	Serine/arginine-rich splicing factor 5	Down	2
SRSF7	Srsf7	Serine/arginine-rich splicing factor 7	Down	2
TXD12	Txndc12	Thioredoxin domain containing 12 (endoplasmic reticulum)	Down	2

Then, the GO biological process and KEGG signaling pathway enrichment analysis were performed separately according to the 30 upregulated key regulatory genes and 13 downregulated key regulatory genes. GL-pp significantly upregulated six main GO biological process clusters, including response to hormone, inositol lipid-mediated signaling, glycerolipid metabolic process, lipid catabolic process, positive regulation of growth, and morphogenesis of a branching epithelium (Figure 5B), and seven KEGG pathways, including focal adhesion, glycerophospholipid metabolism, choline metabolism in cancer, metabolism of xenobiotics by Cyt P450, purine metabolism, extracellular matrix (ECM)–receptor interaction, and cAMP signaling pathway (Figure 5C); in contrast, it significantly downregulated herpes simplex infection and mRNA processing pathways (Figure 5D).

We also performed the same analysis on all the 43 key regulatory genes. While some items were the same as above, some new terms were identified, including valine, leucine, and isoleucine degradation; adenine ribonucleotide biosynthesis; herpes simplex infection; platelet activation; and actin filament–based process (Figure 5E).

The upregulated GO and KEGG terms (Figure 5F) as well as combined terms (Figure 5G) were rendered as network plots to probe the connection between different terms. Visualized by Cytoscape, each node represented an enriched term, and the nodes were colored by clusters. The “focal adhesion” and “response to hormone” clusters had more and tighter correlations to other terms and were situated in the center of the plots. The “focal adhesion” cluster included choline metabolism in cancer (mmu05231), PI3K–Akt signaling pathway (mmu04151), and MAPK signaling pathway (mmu04010), whereas the “response to hormone” cluster included pathways in cancer (mmu05200), small-cell lung cancer (mmu05222), and chemokine signaling pathway (mmu04062). The detailed terms in the two clusters are shown in Table 2. Ptk2, Grb2, and *Lama*2 were included in most terms of “focal adhesion” and “response to hormone” clusters, and the degrees of the three genes were quite high. Especially, the degree of *Lama*2 was the highest among the 43 genes.

**TABLE 2:** Terms of the KEGG signal pathway and GO biology function in “focal adhesion” and “response to hormone” clusters based on the analysis of the global signal transduction network.

cluster	Terms	Description	lgP	Genes
**Focal adhesion**	mmu04510	Focal adhesion	−7.87302	Ptk2, Grb2, *Lama*2, pdgfra, vwf, parva, flnc
	mmu05231	Choline metabolism in cancer	−4.87407	Grb2, pdgfra, Lypla1, Pld1
	mmu04151	PI3K–Akt signaling pathway	−3.84789	Ptk2, Grb2, *Lama*2, pdgfra, vwf
	mmu04014	Ras signaling pathway	−3.46729	Grb2, afdn, pdgfra, Pld1
	mmu04072	Phospholipase D signaling pathway	−2.87998	Grb2, pdgfra, Pld1
	mmu05205	Proteoglycans in cancer	−2.46327	Ptk2, Grb2, flnc
	GO:0061138	Morphogenesis of a branching epithelium	−2.356	Grb2, pdgfra, Yap1
	GO:0030029	Actin filament-based process	−2.28504	Grb2, pdgfra, parva, flnc, Tpm4
	GO:0001763	Morphogenesis of a branching structure	−2.2483	Grb2, pdgfra, Yap1
	mmu04010	MAPK signaling pathway	−2.21937	Grb2, pdgfra, flnc
**Response to hormone**	GO:0009725	Response to hormone	−4.15411	Aldh3a1, Ptk2, Grb2, Pld1, sord, Yap1, Lpin2
	mmu05222	Small cell lung cancer	−3.60391	Ptk2, *Lama*2, nfkbia
	mmu05200	Pathways in cancer	−3.5909	Ptk2, Grb2, *Lama*2, nfkbia, pdgfra
	GO:1901652	Response to peptide	−3.57565	Ptk2, Grb2, nfkbia, Pld1, Lpin2
	mmu05215	Prostate cancer	−3.54359	Grb2, nfkbia, pdgfra
	GO:0043434	Response to peptide hormone	−2.85245	Ptk2, Grb2, Pld1, Lpin2
	mmu04062	Chemokine signaling pathway	−2.54939	Ptk2, Grb2, nfkbia
	GO:0071375	Cellular response to peptide hormone stimulus	−2.23374	Ptk2, Grb2, Lpin2
	GO:0032870	Cellular response to hormone stimulus	−2.11301	Ptk2, Grb2, Yap1, Lpin2
	GO:1901653	Cellular response to peptide	−2.03188	Ptk2, Grb2, Lpin2
	GO:0071417	Cellular response to organonitrogen compound	−2.00286	Ptk2, Grb2, pdgfra, Lpin2

### GL-pp Changed Gut Microbiota Composition in Mice Bearing B16-F10-luc-G5 With SF.

**FIGURE 6:**
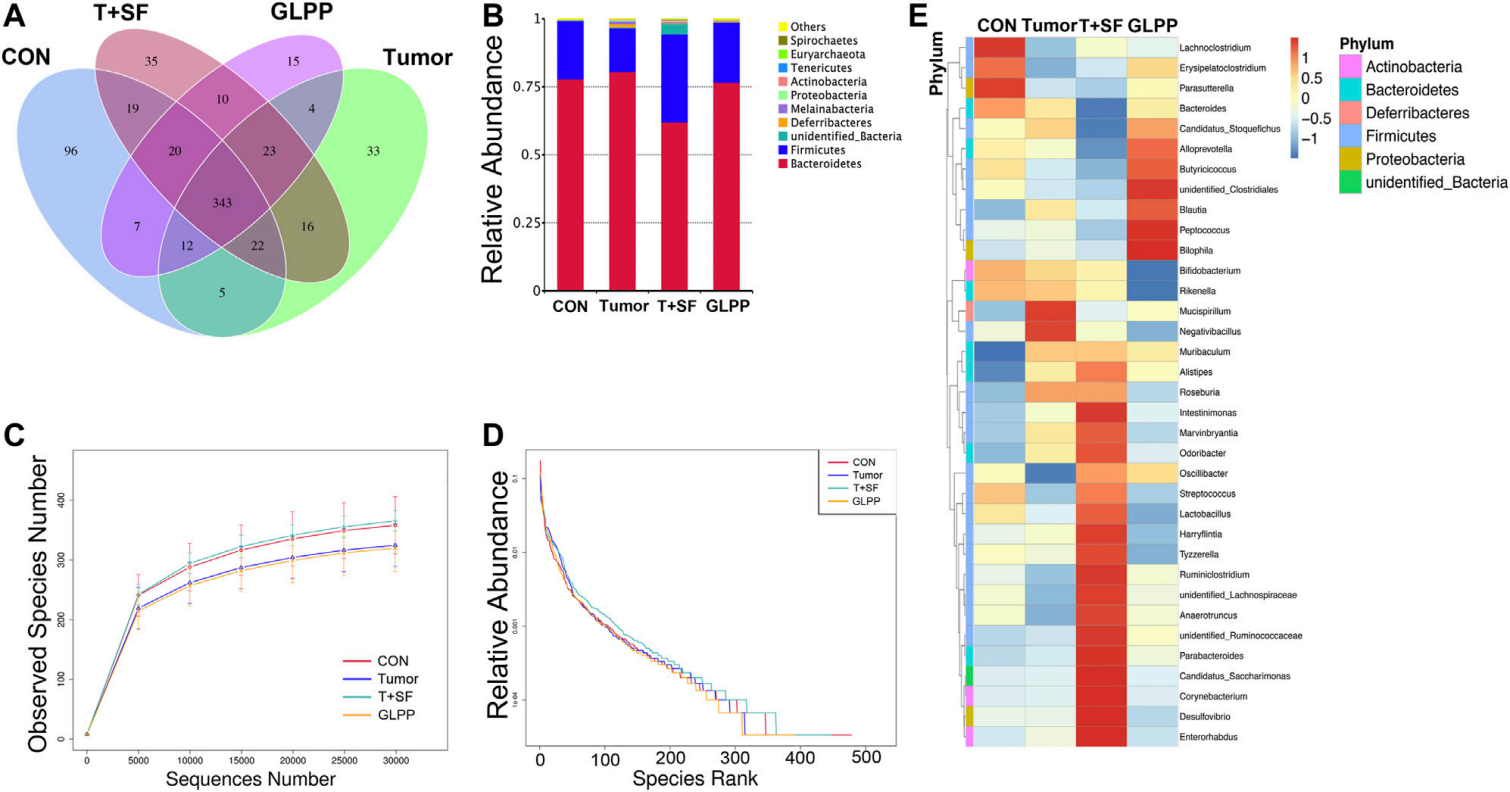
Effect of GL-pp on species number and relative abundance of the gut microbiota in mice with B16-F10-luc-G5 and SF. **(A)** The Venn diagram of OTUs in CON, Tumor, T + SF, and GL-pp groups. **(B)** Relative abundance of the gut microbiota in the four groups at the phylum level. **(C)** The rarefaction curves. **(D)** The relative abundance curves. **(E)** The heat map of top 35 genera of gut microbiota in the four groups with the phylum on the left side (different phyla are shown in different colors).

**FIGURE 7:**
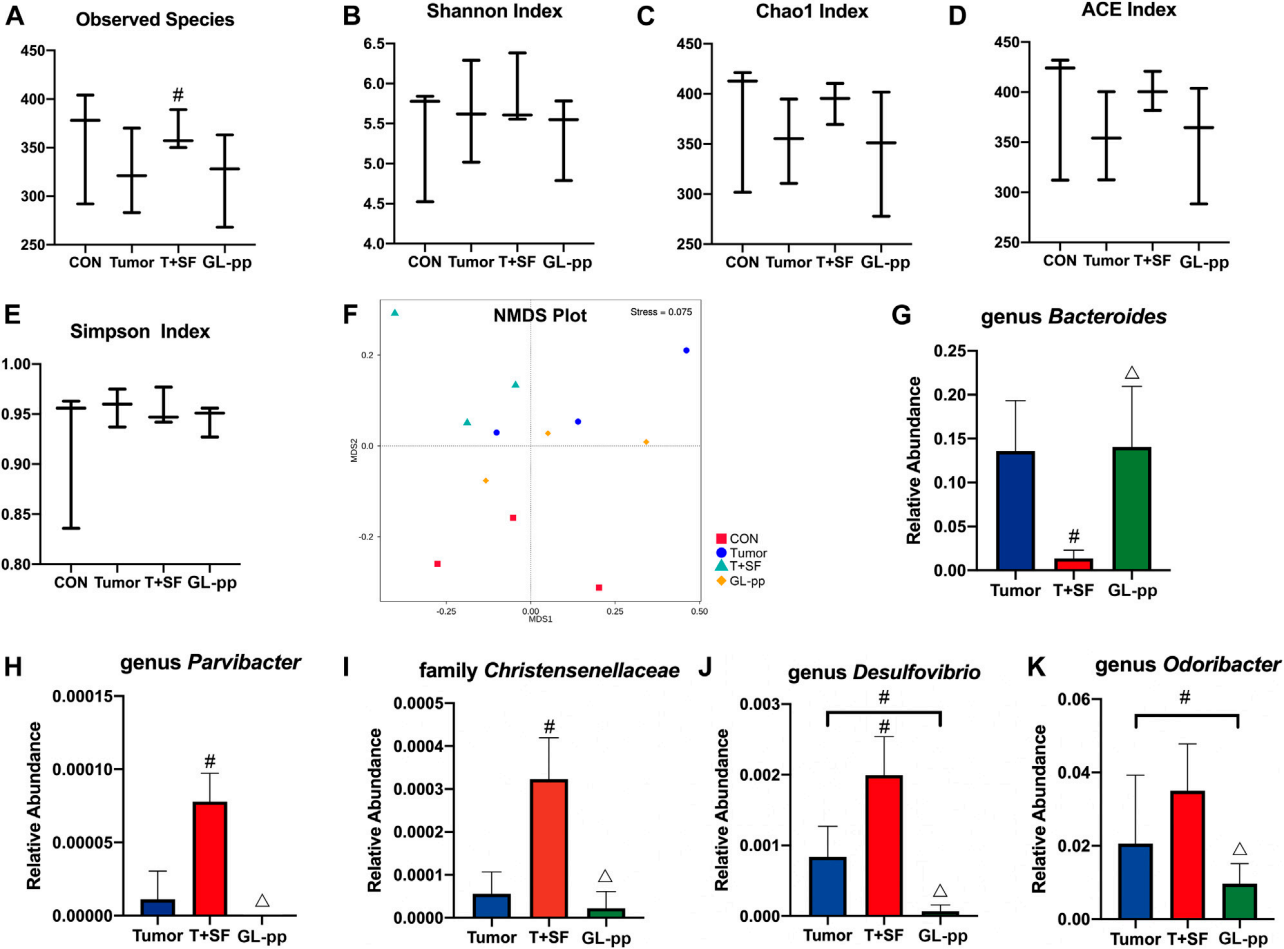
Effect of GL-pp on the diversity and the significantly altered taxa of gut microbiota in mice with B16-F10-luc-G5 and SF. The α-diversity analysis including observed species **(A)**, Shannon Index **(B)**, Chao1 Index **(C)**, ACE Index **(D)**, and Simpson Index **(E)** is shown. The NMDS analysis results are shown in **(F)**. The relative abundance of significantly altered taxa, including genus *Bacteroides* **(G)**, genus *Parvibacter* **(H)**, family *Christensenellaceae* **(I)**, genus *Desulfovibrio* **(J)**, and genus *Odoribacter* **(K)** in the Tumor, T + SF, and GL-pp groups are shown in **(G–K)**. n = 3. Results are shown as mean ± SD. ^#^ *p* < 0.05 compared with the Tumor group; ^△^ *p* < 0.05 compared with the T + SF group.

GL-pp may enhance antitumor immune function, and the intestinal tract is one of the tissues in close contact with the external environment and is an important organ system to defend the body against pathogenic microorganisms. In the present research, the gut microbiota composition was evaluated using the 16S rRNA high-throughput pyrosequencing. Data have been uploaded to the NCBI SRA database (Accession no.: PRJNA680896); 930,027 clean reads were clustered into OTUs under a similarity level of 97%, and the gut microbiota composition was elucidated (Figure 6A). The original sequencing data were uploaded to the NCBI SRA database (Accession no.: PRJNA680896). Most species (343) were the same in the four groups. Especially, the T + SF group and the GL-pp group had 396 taxa in common; the GL-pp group had 38 taxa that were not identified in the T + SF group, including 15 unique taxa of the GL-pp group. The rarefaction curve (Figure 6C) and rank abundance curve (Figure 6D) indicated that the observed species of gut microbiota in the T + SF group increased significantly compared with that in the Tumor group. Consistently, the cluster heat map of species abundance (Figure 6E) and the significant changes of observed species (Figure 7A) indicated that SF altered the composition of the gut microbiota intensively, while the GL-pp administration improved the situation.

Compared with the CON and the Tumor groups, the relative abundance of *Firmicutes* increased significantly in the T + SF group (*p* < 0.05), while the relative abundance of Bacteroidetes decreased, leading to a rise in the Firmicutes:Bacteroidetes (F:B) ratio (*p* < 0.05). After the administration of GL-pp, this situation was significantly reversed to the former level as the CON group’s and the Tumor group’s abundance (*p* < 0.05, Figure 6B). However, the α-diversity analysis, including the Shannon index (Figure 7B), Chao1 index (Figure 7C), ACE index (Figure 7D), and Simpson index (Figure 7E), showed no statistically significant difference in gut microbiota diversity among the four groups, whereas the β-diversity analysis (including PCA, PCoA, and NMDS analysis) indicated a distinct clustering of the samples (Figure 7F).

Furthermore, the significantly altered taxa in the Tumor group, the T + SF group, and the GL-pp group were analyzed using the *t* test, MetaStat, and LDA effect size (LEfSe) method. Five typical taxa were identified: genus *Bacteroides* (phylum Bacteroidetes, Figure 7G), genus *Parvibacter* (phylum Actinobacteria, Figure 7H), family Christensenellaceae (phylum Firmicutes, Figure 7I), genus *Desulfovibrio* (phylum Proteobacteria, Figure 7J), and genus *Odoribacter* (phylum Bacteroidetes, Figure 7K). They were decreased (*Bacteroides*) or increased (*Parvibacter*, *Christensenellaceae*, *Desulfovibrio*, and *Odoribacter*) significantly in the T + SF group (*p* < 0.05), while the levels of *Bacteroides* increased and those of *Parvibacter*, *Christensenellaceae*, *Desulfovibrio*, and *Odoribacter* decreased in the GL-pp group when compared with the levels of those in the T + SF group (*p* < 0.05).

### GL-pp Influenced the Macrophages Polarization and Blood Serum TNF-α Level in Mice Bearing B16-F10-luc-G5 with SF.

**FIGURE 8:**
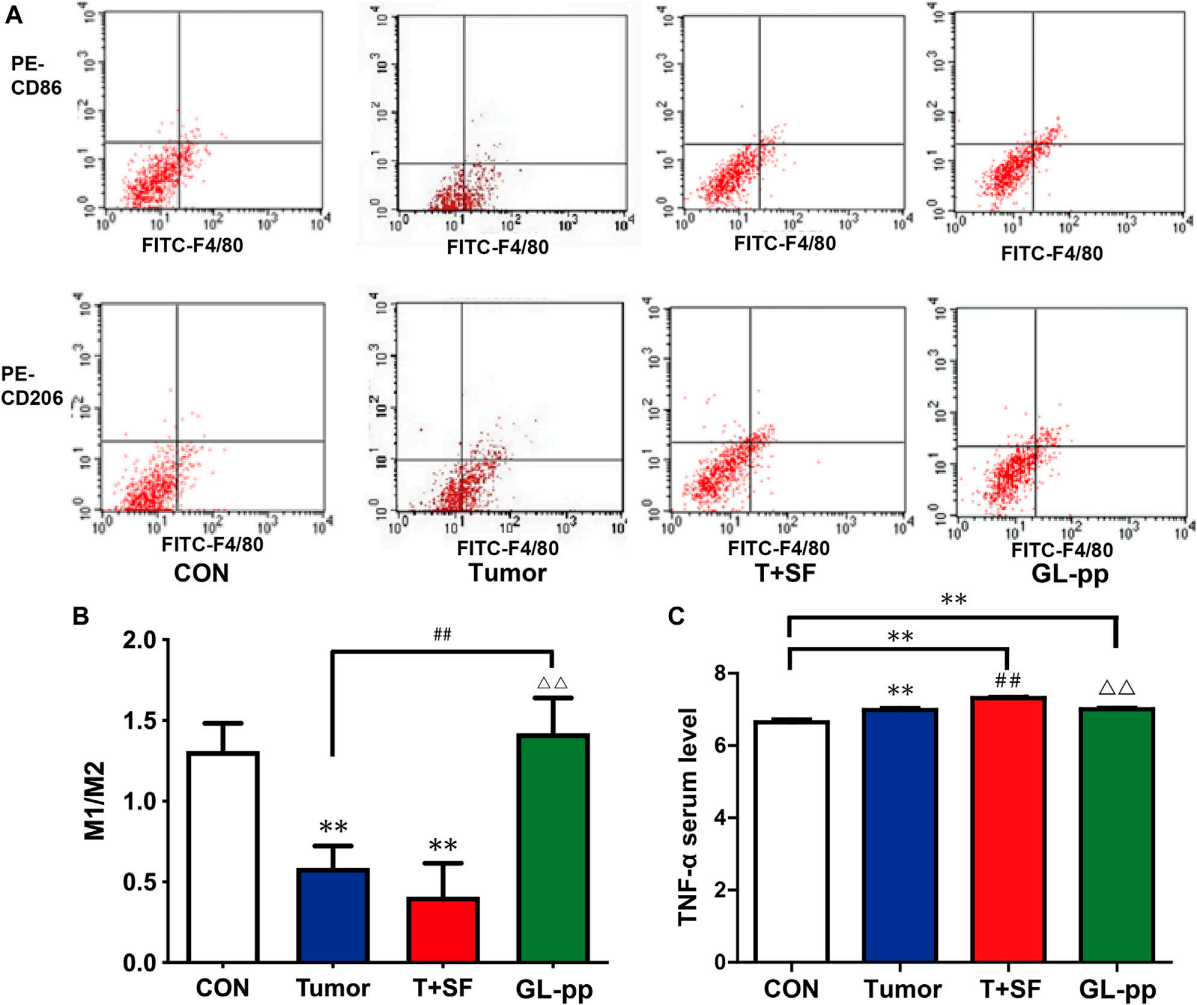
Effect of GL-pp on TAM polarization and TNF-α serum level in mice with B16-F10-luc-G5 and SF. **(A)** The flow cytometry results with CD86 and CD206 label to show TAM polarization. The type of tumor-related macrophages in pulmonary metastasis tumor tissue was detected with CD86 for type M1 macrophage and CD206 for type M2 macrophage. **(B)** The statistical results of M1/M2 ratio. **(C)** The TNF-α serum level in mice detected by ELISA. n = 5. The results are shown as mean ± SD. **p <0.01 compared with the CON group; ^##^ *p* < 0.01 compared with the Tumor group; ^△△^ *p* < 0.01 compared with the T + SF group.

To determine the differentiation direction of macrophages, which existed in tumor environment, we detected the ratio of M1 macrophage to M2 macrophage in the lung tissue of mice bearing B16-F10-luc-G5 with SF. As shown in Figure 8, compared with the CON group, the M1/M2 ratio in both the Tumor group and the T + SF group slightly diminished, indicating a tendency to more polarization to M2 macrophages. The level of serum TNF-α significantly increased in both the Tumor group and the T + SF group, compared with the CON group (*p* < 0.01). The M1/M2 ratio was significantly elevated in the GL-pp group compared with that in the Tumor group or the T + SF group (*p* < 0.01). Consistently, the TNF-α serum level decreased significantly in the GL-pp group compared with that in the Tumor group or the T + SF group (*p* < 0.01).

## Discussion

This study clarified the inhibitory effect of GL-pp on tumor metastasis in mice bearing B16-F10-luc-G5 subjected to SF. First, we confirmed that SF significantly facilitated the lung metastasis in mice bearing B16-F10-luc-G5, which is consistent with previous studies (Cao et al., 2015; Hakim et al., 2014). Next, GL-pp treatment was able to significantly inhibit lung tumor metastasis in mice bearing B16-F10-luc-G5 tumor cells under the SF conditions. GL-pp’s antitumor effects have been studied and shown in several animal tumor models (Ma et al., 2013; Lin and Hsu, 2016). Nevertheless, this is the first time that GL-pp’s antitumor function under the influence of sleep disorder has been clarified.

With the whole proteomics analysis, we found 43 key regulatory genes in lung tissue of mice bearing tumor cells with SF after GL-pp administration. Genes with higher degrees occupied more important positions in the signaling network, and the degree of *Lama2* was the highest among the 43 genes when analyzed by the global signal transduction network, indicating a strong correlation between *Lama2* and other genes in the signal transduction network. Downregulation of *Lama2* expression was also found, notably in liver, ovarian, lung, and colorectal cancer (Jhunjhunwala et al., 2014). In laryngeal squamous cell carcinoma (Ni et al., 2012) and breast cancer (Mefford and Mefford, 2012), downregulation of *Lama2* expression was linked to tumor progression. Previous studies found that a decrease in *Lama2* expression was accompanied by an increase in DNA methylation near the transcription start site; promoter DNA methylation and downregulation across multiple cancer types indicate that *Lama2* is a tumor suppressor gene (Wang et al., 2019).

The GO and KEGG analyses revealed that focal adhesion and response to hormone clusters had more and tighter correlations to other terms and were situated in the center of the plots. Focal adhesion, in which focal adhesion kinase (FAK) is overexpressed in cancer cells and tumor microenvironment, has been shown to improve tumor progression and metastasis (Sulzmaier et al., 2014). Lin TY et al. found that one kind of *G. lucidum* recombinant protein inhibited the epithelial to mesenchymal transition (EMT) process by disturbing the function of cell adhesion and focal adhesion kinase (FAK) in lung cancer cells (A549 and CL1-5 human NSCLC adenocarcinoma cell lines and LLC1 Lewis lung carcinoma cell line) (Lin and Hsu, 2016; Sun and Sun, 2019).

Ptk2 and Grb2 are important genes participating in the pathway of focal adhesion, and we found that the degrees of the two genes were quite high. Overexpression of Ptk2, which encodes FAK, improves cell migration, invasion, adhesion, proliferation, and survival in ovarian and other cancers (Levy et al., 2019). Growth factor receptor–bound protein 2 (Grb2) has been linked to many oncogenic pathways. For example, in breast cancer, Grb2 links with SHP1 to proceed tumor progression. In non–small-cell lung cancer, Grb2 is involved in tumor metastasis by regulating both MAPK and Akt pathways (Ijaz et al., 2018). According to our experiment and previous studies, we speculate that GL-pp might inhibit tumor metastasis by disturbing the function of FAK and the pathway involving Ptk2 and Grb2.

Recently, the correlation among gut microbiota, inflammation, and cancer has been widely considered (Morgillo et al., 2018). Under normal conditions, the gut microbiota and gastrointestinal environment are in a mutually beneficial relationship (Lozupone et al., 2012). However, a lot of evidence has indicated that gut microbiota has a strong influence on oncogenesis, tumor progression, and response to therapy (Zitvogel et al., 2015). For example, gut microbiota can significantly affect tumor growth in mice (Sethi et al., 2018), contribute to antitumor immunity, limit tumor expansion (Li et al., 2019), and significantly influence the efficacy of immunotherapy (Routy et al., 2018). These findings highlighted the importance to investigate the alterations in the gut microbiota.

As expected, the 16S rRNA sequencing indicated that the Firmicutes:Bacteroidetes (F:B) ratio significantly increased in tumor-bearing mice subjected to SF; after the administration of GL-pp, the ratio decreased significantly compared with the T + SF group. Notably, the F:B ratio is considered an indicator of structural modifications (Poroyko et al., 2016), and the increased F:B ratio is associated with inflammation (Ismail et al., 2011) and poor prognosis of many diseases (Li et al., 2017; Spychala et al., 2018). As for the specific taxa, our results showed an antagonizing effect of GL-pp against tumor under SF conditions. The genus *Bacteroides*, which has an immunosuppressive function (Zhang et al., 2018), was significantly downregulated in the T + SF group and upregulated in the GL-pp group. *Desulfovibrio*, a kind of opportunistic pathogens, has been related to some inflammatory diseases (Cani et al., 2012) through H_2_S-related damage of the gut epithelium energy metabolism (Babidge et al., 1998; Cao et al., 2019). Our results also showed a significant decrease of *Desulfovibrio* in the GL-pp group compared with both the T + SF and the Tumor groups. As for Christensenellaceae and *Odoribacter*, Mancabelli L et al. showed that Christensenellaceae could be one of the specific disease microbial biomarkers for colorectal cancer when compared with control subjects (Mancabelli et al., 2017). Zackular JP et al*.* demonstrated that the destructive changes in the gut microbiome were closely related to the development of colorectal cancer, and mice with intestinal colonization of intestinal microflora among tumor-bearing mice had a higher risk of tumorigenesis. Tumor-bearing mice showed enrichment of *Odoribacter* genera and others, and antibiotic treatment of these bacteria was shown to reduce the number and size of tumors (Zackular et al., 2013). These findings indicate a possible immunoregulative effect of GL-pp, which enhances the intestinal barrier function. Therefore, GL-pp might achieve the antitumor effect partially through altering the gut microbiota.

In our present study, we used immunecompromised Balb/c athymic nude mice to observe the lung metastasis effect of B16F10 under the IVIS luminescent system. We could deduce that GL-pp had inhibitory effects on SF-promoted tumor metastasis even under the circumstances of T-cell deficiency.

Tumor-associated macrophages (TAMs) are a cluster of immune cells observed in the microenvironment of solid tumors; they have two principal phenotypes (M1 and M2) (Komohara et al., 2016). At present, M1 is mostly related to the inflammatory response and antitumor immunity, whereas M2 is associated with immunosuppressive functions and tumor growth promotion (Chanmee et al., 2014). Clinically, cancer patients with a higher M1/M2 ratio displayed a better prognosis and overall survival rate (de la Cruz-Merino et al., 2013; Williams et al., 2016). Hakim et al. (2014) exposed a group of mice to chronic SF, mimicking several sleep disorders; they observed significant increases in TAM counts in tumor tissue compared with the controls. They also found that SF induced TAM polarity shift toward M2 (Hakim et al., 2014). Our results also showed the decrease in the M1/M2 ratio in the Tumor and the T + SF groups. Our findings also showed that GL-pp at the dose of 80 mg/kg was able to significantly increase the M1/M2 ratio in lung metastasis tumor of mice with SF. Clearly, GL-pp increased the tendency of macrophage polarization from M2 to M1 in TAMs *in vivo*, indicating an antitumor effect of GL-pp.

Meanwhile, we showed that the serum TNF-α level increased in tumor-bearing mice subjected to SF; after the administration of GL-pp, the serum TNF-α level decreased. Some studies found that TNF-α was increased by the *Ganoderma lucidum* extract complex in the tumor-bearing mice serum (Zhao et al., 2018b); polysaccharides from fresh fruiting bodies of *Ganoderma lucidum* were also able to greatly increase the TNF-α level in macrophage culture cells (Wang et al., 1997). Interestingly, other studies found that triterpene ganoderic acid C1 isolated from *Ganoderma lucidum* downregulated TNF-α production by macrophages and peripheral blood mononuclear cells (PBMCs) from Crohn’s disease subjects (Liu et al., 2015). *Ganoderma lucidum* also reduced the TNF-α level under the inflammatory conditions (Dudhgaonkar et al., 2009); inflammation is frequent in some kinds of cancers and is known to facilitate the growth and metastasis of tumor cells (Ali et al., 2018). Increased TNF-α is related to cancer cachexia, which is manifested by body weight loss, chronic nausea, fatigue, insomnia, and profuse sweating; in contrast, the reduction in TNF-α correlates with improved fatigability in cancer patients (Argilés and López, 1999; Mendes et al., 2015). GL-pp can also be used to treat chemotherapy-related fatigue and improve the quality of life of individuals by lowering the serum levels of TNF-α that were upregulated in patients undergoing chemotherapy (Lin and Sun, 2019). Therefore, the reduction of TNF-α observed in the current study may imply an anti-inflammatory effect of GL-pp.

## Conclusion

Our study suggested that GL-pp had an antitumor metastasis effect under the SF conditions. Moreover, GL-pp affected the proteomics profile, gut microbiota composition, macrophage polarization, and TNF-α level in mice bearing B16-F10-luc-G5 with SF. We believe that the present study indicates a prospect for *Ganoderma lucidum* to be further studied and used widely in the future.

## Data Availability

The datasets presented in this study can be found in online repositories. The names of the repository/repositories and accession number(s) for microbiota data can be found below: https://www.ncbi.nlm.nih.gov/sra/PRJNA680896, and the mass spectrometry proteomic data can be found in the ProteomeXchange Consortium *via* the PRIDE partner repository with the dataset identifier PXD025171.
