# Supplementary material for: Antimetastatic Effects of Ganoderma lucidum Polysaccharide Peptide on B16-F10-luc-G5 Melanoma Mice With Sleep Fragmentation
Source: Front Pharmacol. 2021 Jul 8;12:650216. doi: 10.3389/fphar.2021.650216 (PMC8296642; doi:10.3389/fphar.2021.650216)
Supplement: Supplementary file 1 [file Table1.pdf]

**Table S1 Two hundred and twenty-seven differentially expressed genes in lung metastasis tissue in mice treated with GL-pp group compared with T+SF group.**

| **Gene Symbol** | **Majority Protein IDs** | **-Lg*P*** |
|---|---|---|
| Iqsec1 | E9PUA3;A0A087WQ39;Q8R0S2;Q8R0S2-2 | 1.46174 |
| Nudt16 | A0A087WRE5;Q6P3D0;A0A087WSB5 | 1.4719 |
| Arhgap21 | Q6DFV3;B7ZCJ0;A0A0A0MQE8;B7ZCJ1;G3UWM5 | 1.47788 |
| Nptn | A0A0A0MQN8;H3BIX4;Z4YLB7;P97300-3;P97300-1;P97300 | 1.4315 |
| Rab3gap2 | Q8BMG7;A0A0A6YWM5;E9QKE4 | 2.13792 |
| Mast4;Mast2 | E9PWX8;E9QPR4;A0A0A6YXG1;Q811L6;E9Q6G0;E9Q4Q9;Q811L6-2;E9Q1M8;B1AST8;Q60592;B1AST7;E9QLW6;E9Q1Q1 | 1.54809 |
| Kiaa1522 | A2A7S8-2;A2A7S8-3;A2A7S8;A2A7S8-4 | 2.04034 |
| Dock11 | A2AF47;A2AF67 | 2.27421 |
| Ubr4 | A2AN08-3;A2AN08;A2AN08-5 | 1.31362 |
| Aqr | A2AQA7;Q8CFQ3 | 1.6308 |
| Tp53bp1;Trp53bp1 | P70399;A2AU91;P70399-3;A2AU89;P70399-2 | 1.96478 |
| Dab2ip | Q3UHC7-2;F6RK07;A2AUX5;Q3UHC7-4;A2AUX3;Q3UHC7-3;B7ZD29;Q3UHC7;F6QQP6 | 1.57137 |
| Dennd3 | A2RT67;G3UYA1;F7B6S5 | 1.46691 |
| Ttc38 | A3KMP2;A3KMP2-2 | 2.82207 |
| Chp1 | B0R091;P61022 | 1.88011 |
| Ssbp3 | Q9D032-2;B1AS37;Q9D032 | 3.18845 |
| Grb2 | B1AT92;Q60631;Q60631-2 | 2.57596 |
| Hgs | B1ATZ0;B1ATZ1;Q99LI8;Q3UMA3;B1ATY9 | 1.54796 |
| Aifm1 | B1AU25;Q9Z0X1 | 2.37571 |
| Mtm1 | B1AW21;Q9Z2C5 | 1.7601 |
| Dock5 | B2RY04 | 1.37987 |
| Flnc | Q8VHX6-2;D3YW87;Q8VHX6;D3Z576 | 1.36591 |
| Tmem109 | D3Z7P2;D3Z3Z2;D3YX08;D3Z0I8;Q3UBX0 | 1.52107 |
| Snrk | D3Z1F6;Q8VDU5 | 2.29587 |
| Fip1l1 | Q9D824-4;D3Z619;Q9D824-3;Q9D824-2;Q9D824;D3Z3F1;D3Z4V2 | 1.77142 |
| Pld1 | Q9Z280;D6RH77;Q9Z280-2;Q6NVF2;F6WKY8 | 1.63769 |
| Mob4 | E0CXA9;Q6PEB6;E0CYJ0;G8JL35 | 1.34826 |
| Urb1 | Q571H0;E9PU96 | 1.73503 |
| Csnk1a1 | E9Q4G7;Q8BK63;E9Q3W1;E9PWB2;Q8BK63-2;H7BXB1;E9Q2U6;F6YBC9 | 1.39774 |
| Lpin2 | Q99PI5;E9PWN0 | 2.28276 |
| Itsn1 | Q9Z0R4;E9Q0N0;E9Q3I4;Q9Z0R4-2;E9Q3I5;E9Q3I8;E9Q3I9 | 1.55464 |
| Gm15800 | E9Q2E4;E9PX61 | 1.8388 |
| Pi4ka | E9Q3L2 | 1.69168 |
| Kif14;kif14 | E9Q3T3;L0N7N1 | 2.32863 |
| Mvp | Q9EQK5;E9Q3X0 | 2.06793 |
| Nbas | E9Q411 | 2.30592 |
| Dctn1 | E9Q586;O08788-2;E9Q3M3;O08788;D3YX34 | 1.58502 |

| | | |
|---|---|---|
| Myo1e | E9Q634 | 1.37397 |
| Mllt4 | E9Q9C3 | 1.36837 |
| Ccdc93 | E9QAD4;Q7TQK5 | 1.37062 |
| Chd4 | E9QAS4;Q6PDQ2;E9QAS5 | 1.47152 |
| Shroom3 | Q9QXN0-3;E9QMY5;Q9QXN0-4;Q9QXN0;Q9QXN0-2 | 1.34679 |
| Itsn2 | E9QNG1;Q9Z0R6;Q9Z0R6-2 | 1.90506 |
| Vwf | Q8CIZ8;E9QPU1 | 1.70974 |
| Col18a1 | P39061-2;P39061-1;P39061;E9QPX1 | 2.14233 |
| Iqce | F6R782;Q6PCQ0-2;Q6PCQ0 | 1.45197 |
| Ctss | O70370;F6WR04 | 1.56976 |
| Spef1 | F6ZW13;Q99JL1 | 1.57958 |
| Agap3 | Q8VHH5;F8VQE9;Q8VHH5-2 | 1.52072 |
| Sash1 | P59808;F8VQK5 | 1.90949 |
| Sftpb | S4R2L6;F8WGK6;P50405;S4R1N7;S4R239 | 2.08077 |
| Ddx24 | Q9ESV0;F8WJA0 | 1.32537 |
| Zc3h18 | Q0P678;G3X8T2;H3BIW0;H3BJJ9 | 2.066 |
| Ctsa | P16675;G3X8T3 | 1.45249 |
| Dnm2 | G3X9G4;Q3TCR7 | 1.33661 |
| Ckap5 | K3W4R5;Z4YL78;A2AGT5-3;A2AGT5;A2AGT5-2 | 1.65777 |
| Serpinb6b | O08804;F7B9A0 | 2.60254 |
| Cyth3 | O08967;G5E8Q4 | 1.63327 |
| Srsf5 | O35326;Q9D8S5 | 1.41643 |
| Anxa8 | O35640;Q921D0 | 1.87681 |
| Sptlc1 | O35704 | 1.38061 |
| Gp1ba | O35930 | 2.04769 |
| Ddost | O54734 | 2.13331 |
| Rpl35a | O55142 | 1.83453 |
| Mmp8 | O70138 | 1.60971 |
| Hmox2 | O70252;D3YX62 | 1.37959 |
| Sorl1 | O88307 | 2.05796 |
| Plin4 | O88492-2;O88492 | 1.61097 |
| Mb | P04247 | 1.3543 |
| H1f0 | P10922 | 1.87662 |
| Umps | P13439 | 1.45934 |
| Hspb1 | P14602;P14602-2;D3YZ06;P14602-3 | 1.50879 |
| Hist1h1c | P15864 | 1.47504 |
| Mut | P16332 | 1.83928 |
| Ubl4a | P21126 | 1.47862 |
| Tgm2 | P21981 | 1.4393 |
| Pdgfra | P26618;P26618-2 | 1.45752 |
| Rpl3 | P27659 | 1.33626 |
| Adssl1 | P28650;P28650-2;J3QN31 | 3.40216 |
| Kras | P32883-2 | 1.87109 |

| | | |
|---|---|---|
| Ptk2 | P34152-3;P34152-4;P34152-2;P34152;P34152-6;P34152-5 | 1.41824 |
| Csk | P41241 | 2.33919 |
| Hist1h1b | P43276 | 1.66268 |
| Adss | P46664 | 1.86516 |
| Yap1 | P46938-2;P46938;G3UYA6;G3UY62;G3UYV4;G3UYW7 | 1.30736 |
| Aldh3a1 | P47739 | 1.881 |
| Lmna | P48678;P48678-2;P48678-3 | 1.60338 |
| Hsd17b8;H2-Ke6 | P50171;P50171-2;G3UX44 | 1.7054 |
| Usp10 | P52479-2;P52479 | 1.37132 |
| Nub1 | P54729 | 1.4769 |
| Cox6b1 | P56391 | 1.83554 |
| Rin3 | P59729;Q3U332 | 1.33989 |
| Magoh;Magohb | P61327;Q9CQL1;A0A023T778;G3UZW7;G3UYI5 | 1.34429 |
| Copz1 | P61924 | 1.50386 |
| Rps8 | P62242 | 1.61337 |
| Rpl18a | P62717 | 1.47665 |
| Mtpn | P62774 | 1.7234 |
| Hist1h4a | P62806 | 1.48535 |
| Rpl30 | P62889 | 1.58856 |
| Rpl32 | P62911 | 1.80634 |
| Rpl8 | P62918 | 1.7102 |
| Crnkl1 | P63154 | 1.55864 |
| Rps17 | P63276 | 1.5743 |
| Stim1 | P70302 | 1.52051 |
| Slc9a3r1 | P70441 | 1.37481 |
| Cct5 | P80316 | 1.36414 |
| Fmo3 | P97501 | 1.38488 |
| Lypla1 | P97823;P97823-2;J3QP56;D3YUG4;D3Z111 | 1.44748 |
| Cab39 | Q06138 | 1.40014 |
| Dnajb2 | Q9QYI5-2;Q9QYI5;Q3TB24 | 1.56135 |
| Hp1bp3 | Q3TEA8-2;Z4YKB8;Q3TEA8;Z4YKA3;Q3TEA8-3;A2AM65 | 1.48437 |
| Lpcat1 | Q3TFD2;Q3TFD2-2;Q3TFD2-3 | 1.56185 |
| Pde12 | Q3TIU4 | 2.10927 |
| Zc3h15 | Q3TIV5 | 1.45454 |
| Ap5z1 | Q3U829 | 1.425 |
| Lbr | Q3U9G9;A0A0A6YY12 | 3.38316 |
| Parva | Q3UF75;Q9EPC1 | 1.42049 |
| Plekha7 | Q3UIL6-3;Q3UIL6-4;Q3UIL6-5;Q3UIL6;Q3UIL6-6;Q3UIL6-2 | 1.31966 |
| Sf3b2 | Q3UJB0 | 1.50682 |
| Parp3 | Q8CFB8;Q3ULW8 | 1.35497 |
| Nme7 | Q8BUH2;Q9QXL8;Q3UMG6 | 2.04517 |
| Iqgap2 | Q3UQ44 | 1.89883 |
| Rcsd1 | Q3UZA1-2;Q3UZA1 | 1.44466 |

| | | |
|---|---|---|
| Phactr4 | Q501J7-2;Q501J7;Q501J7-3 | 1.5586 |
| Thrap3 | Q8BZN7;Q569Z6 | 1.3066 |
| Lrrk2 | Q5S006 | 1.81123 |
| Tom1l2 | Q5SRX1;Q5SRX1-2;Q5SRX1-3;Q5SRX1-4;Q5SXA5;Q5SXA4 | 1.41588 |
| Clint1 | Q5SUH6;Q5SUH7;Q99KN9-2;Q99KN9 | 2.24784 |
| Lama2 | Q60675;A0A087WRP2 | 1.4191 |
| Aplp2 | Q60709;Q06335-2;Q06335 | 1.76368 |
| Stxbp3 | Q60770;Q60770-2 | 1.38385 |
| Pml | Q60953;D3Z3A6;D3YXR5;Q60953-2;F7BTZ2 | 2.93636 |
| Hcfc1 | Q61191;B1AUX2 | 1.70043 |
| Arhgdib | Q61599 | 1.5929 |
| Kif5b | Q61768;E9QAK5 | 1.86392 |
| Mrc1 | Q61830 | 1.37078 |
| Aebp1 | Q640N1;Q640N1-2 | 1.73289 |
| Sord | Q64442 | 1.75467 |
| Tpm4 | Q6IRU2 | 1.33851 |
| Rrp12 | Q6P5B0 | 1.33256 |
| Kif15 | Q6P9L6 | 1.36106 |
| Fkbp15 | Q6P9Q6;Q6P9Q6-2;Q80YW9 | 1.33296 |
| Nostrin | Q6WKZ7 | 2.0936 |
| Kctd12 | Q6WVG3 | 1.52664 |
| Acap2 | Q6ZQK5;Q6ZQK5-2 | 1.44803 |
| Rpl36 | Q6ZWZ4 | 1.43883 |
| Fbxo22 | Q78JE5;Q3V492 | 2.19012 |
| Fam98b | Q80VD1 | 1.31913 |
| Aldh3b1 | Q80VQ0 | 1.42528 |
| Crybg3 | Q80W49;Q80W49-2;Q80W49-3 | 2.6132 |
| Tbc1d10b | Q8BHL3 | 1.33971 |
| Srsf7 | Q8BL97-4;Q8BL97-2;Q8BL97;Q8BL97-3 | 1.505 |
| Ckap4 | Q8BMK4 | 1.48036 |
| Hadha | Q8BMS1 | 1.4617 |
| Metap1 | Q8BP48 | 1.78691 |
| Heatr3 | Q8BQM4;E9PWH6 | 1.31314 |
| Cpsf7 | Q8BTV2-2;Q8BTV2;Q8BTV2-3 | 2.58635 |
| Pdcl3 | Q8BVF2 | 1.87185 |
| Zhx2 | Q8C0C0 | 1.32492 |
| Sipa1l1 | Q8C0T5-2;Q8C0T5 | 1.34859 |
| Rbm14 | Q8C2Q3;E9QL13;Q8C2Q3-2 | 2.37903 |
| Nemf | Q8CCP0;Q8CCP0-3;Q8CCP0-2;Q8CCP0-4 | 1.63093 |
| Uaca | Q8CGB3;Q8CGB3-3;Q8CGB3-2 | 1.4111 |
| Sltm | Q8CH25-2;Q8CH25 | 1.63984 |
| Gmds | Q8K0C9 | 1.4818 |
| Alkbh3 | Q8K1E6;A2AKV6;A2AKV7 | 1.64407 |

| | | |
|---|---|---|
| Spats2 | Q8K1N4;Q8K1N4-2 | 1.58534 |
| ptplad1 | Q8K2C9 | 1.62078 |
| Ndufs8 | Q8K3J1 | 1.57 |
| Treml1 | Q8K558;Q8K558-2 | 1.47488 |
| Rab11fip5 | Q8R361 | 2.09643 |
| Nup35 | Q8R4R6;A2ATJ2 | 1.35718 |
| Luzp1 | Q8R4U7 | 2.18108 |
| Gstm4 | Q8R5I6;A2AE91 | 1.66118 |
| Hpgd | Q8VCC1 | 1.3219 |
| Sirt2 | Q8VDQ8-2;Q8VDQ8;Q8VDQ8-4;Q8VDQ8-3 | 2.3846 |
| Lmcd1 | Q8VEE1 | 1.3299 |
| Wdr13 | S4R225;Q91V09 | 1.66899 |
| Memo1 | Q91VH6 | 1.89924 |
| Rnh1 | Q91VI7 | 1.43073 |
| Setd3 | Q91WC0;F2Z420;F2Z438;Q91WC0-2 | 1.31814 |
| Dnajc9 | Q91WN1 | 1.32588 |
| Golm1 | Q91XA2 | 1.35313 |
| N/A | Q91Z58 | 1.38409 |
| Srgap2 | Q91Z67;A0A087WNM1;A0A087WSQ1 | 1.96632 |
| Pcca | Q91ZA3 | 1.52351 |
| Als2 | Q920R0 | 1.57499 |
| Plrg1 | Q922V4;D3Z4V1;F8WI31 | 1.46748 |
| Hadhb | Q99JY0 | 1.30781 |
| Vwa5a | Q99KC8 | 1.35828 |
| Prpf19 | Q99KP6;Q99KP6-2;Q99KP6-3 | 1.51862 |
| Mtfr1 | Q99MB2;D3YZ89 | 2.19893 |
| Vimp | Q9BCZ4 | 1.39722 |
| Mgst3 | Q9CPU4 | 1.7665 |
| Ndufa2 | Q9CQ75 | 1.82202 |
| Rpl21 | Q9CQM8;O09167 | 1.35673 |
| Txndc12 | Q9CQU0 | 1.65926 |
| Rer1 | Q9CQU3 | 1.34536 |
| Ndufb3 | Q9CQZ6 | 1.3097 |
| Ndufab1 | Q9CR21;F6ZFT1;F8WJ64 | 1.48671 |
| Gdpd1 | Q9CRY7 | 1.91877 |
| Pcbd2 | Q9CZL5 | 2.51889 |
| Tsfm | Q9CZR8 | 1.58803 |
| Tmed10 | Q9D1D4 | 1.49567 |
| Tbcb | Q9D1E6 | 1.9512 |
| Mrpl19 | Q9D338 | 1.34735 |
| Rpl22l1 | Q9D7S7-2;Q9D7S7 | 1.67329 |
| Ccdc91 | Q9D8L5 | 2.30968 |
| Slirp | Q9D8T7;Q9D8T7-2;F8WHU8 | 2.88711 |

| | | |
|---|---|---|
| Selo | Q9DBC0;S4R1U9 | 1.81485 |
| Ftsj3 | Q9DBE9 | 2.15269 |
| Twsg1 | Q9EP52 | 1.84058 |
| Nek7 | Q9ES74 | 1.47976 |
| Dkc1 | Q9ESX5;B7ZCL7 | 1.44913 |
| Ccdc22 | Q9JIG7 | 1.65908 |
| Sh3bgrl | Q9JJU8 | 2.68702 |
| Pin1 | Q9QUR7 | 1.9669 |
| Naga | Q9QWR8 | 2.24772 |
| Pkp3 | Q9QY23;Q9QY23-2 | 1.72004 |
| 6-Sep | Q9R1T4-2;Q9R1T4-3;Q9R1T4 | 1.54808 |
| Skp1 | Q9WTX5 | 1.48793 |
| Pdcd6ip | Q9WU78;Q9WU78-3 | 1.37526 |
| Fam50a | Q9WV03 | 1.39758 |
| Snx1 | Q9WV80 | 1.63159 |
| Timm8a1 | Q9WVA2 | 1.61501 |
| Nfkbia | Q9Z1E3 | 1.35614 |
| Abhd16a | Q9Z1Q2 | 2.29504 |
